# Centralized HIV Program Oversight: An Investigation of a Case Series of New HIV Infections among US Army Soldiers, 2012 to 2013: Erratum

**DOI:** 10.1097/01.md.0000479950.17284.98

**Published:** 2016-01-22

In the article “Centralized HIV Program Oversight: An Investigation of a Case Series of New HIV Infections among US Army Soldiers, 2012 to 2013”,^1^ which appeared in Volume 94, Issue 46 of *Medicine*, a typographical error occurred in the abstract. The article has since been corrected online.
